# Instilled air promotes lipopolysaccharide-induced acute lung injury

**DOI:** 10.3892/etm.2014.1523

**Published:** 2014-02-06

**Authors:** YINGGANG ZOU, CHUNLING DONG, MINGZHEN YUAN, GUANGYUAN GAO, SIYI WANG, XIAODING LIU, HUIQIAO HAN, BO LI

**Affiliations:** 1 Department of Human Anatomy, College of Basic Medical Sciences, Jilin University, Changchun, Jilin 130021, P.R. China; 2 Department of Obstetrics and Gynecology, Second Hospital, Jilin University, Changchun, Jilin 130041, P.R. China; 3 Department of Respiratory Medicine, Second Hospital, Jilin University, Changchun, Jilin 130041, P.R. China

**Keywords:** acute lung injury, lipopolysaccharide, intratracheal instillation, instilled air

## Abstract

Optimization of intratracheal instillation is necessary to establish an ideal animal model of acute lung injury (ALI) in order to further reveal the cellular and molecular pathogenesis of ALI. It is possible that instilling air from a prefilled syringe may promote the delivery of reagents into the alveolar spaces, resulting in different pulmonary responses. In the present study, the influence of instilling air by trans-tracheal intratracheal instillation in a lipopolysaccharide (LPS)-induced mouse model of ALI was investigated. The bronchoalveolar lavage (BAL) fluid biochemical index, BAL fluid differential cell counts, lung wet/dry weight ratio, lung histology and BAL fluid interleukin-8 (IL-8) levels were assessed 24 h subsequent to intratracheal instillation. Instilled air promoted LPS-induced ALI, as indicated by the severity of acute pulmonary inflammation and increased IL-8 release. In conclusion, this study indicates that instilled air may be used to improve the intratracheal instillation procedure and to establish a more reliable animal model of ALI.

## Introduction

Acute lung injury (ALI) and its more severe form, acute respiratory distress syndrome (ARDS), are clinical syndromes of acute hypoxic respiratory failure resulting from a variety of direct and indirect injuries to the parenchyma of the lungs (1–3). The mortality rate in patients with ALI/ARDS is ~40% due to slow progress in understanding the mechanisms responsible for disease pathogenesis (4–6). Lipopolysaccharide (LPS) induces symptoms in animal models that closely resemble ALI/ARDS in humans, highlighting strategies to explore the pathogenesis of ALI/ARDS (7–9).

A reliable experimental animal model is necessary to elucidate the cellular and molecular pathogenesis of ALI/ARDS (10–12). In a previous study of mouse models of LPS-induced ALI, two methods of intratracheal instillation were compared (13). LPS-induced pulmonary inflammatory responses were more severe following trans-tracheal intratracheal instillation relative to those following trans-oral intratracheal instillation, which may be due to more effective delivery of LPS to the lungs by trans-tracheal intratracheal instillation. Of note, delivery of LPS with air from a prefilled syringe was associated with rapid instillation into the lung when using the modified procedure of trans-tracheal intratracheal instillation. Therefore, it is possible that instilled air may promote delivery of LPS into the alveolar spaces, resulting in different pulmonary responses. Instilling air from a prefilled syringe has been used to investigate the pulmonary toxicity of nanoparticles (14). However, the influence of intratracheal air instillation on experimental animal models of ALI has not been assessed.

In the present study, the influence of trans-tracheal intratracheal air instillation on a mouse model of LPS-induced ALI was investigated. The aim of the study was to reveal the role of intratracheal air instillation in LPS-induced experimental animal models of ALI.

## Materials and methods

### Animals

Male C57BL/6 mice (n=75; weight, 20±2 g) were purchased from the Jilin University Animal Center (Changchun, China). The animals were housed in a room at 22°C with a 12-h light/dark cycle (6:00 a.m.–6:00 p.m. light). Mice were fed standard mouse chow and provided water *ad libitum*. All animal experiments were approved by the Animal Care Committee of Jilin University.

### Intratracheal instillation

The procedure for trans-tracheal intratracheal instillation was modified from a previous experiment (15). Briefly, mice were anesthetized by intraperitoneal injection of 0.1 ml pentobarbital sodium (50 mg/kg; Sigma, St. Louis, MO, USA) and placed in a supine position head-up on a board. The board was tilted at a 50-degree angle. A midline incision was made in the neck to expose the trachea. LPS (Sigma) was dissolved in 0.9% normal saline (NS) at a concentration of 1 mg/ml. LPS at a dosage of 5 mg/kg or the same volume of NS was drawn into a sterile plastic catheter (up to a premarked level) through a 29-gauge needle and rapidly instilled into the lung with a 1-ml syringe. Following intratracheal instillation, the mice were placed vertically and rotated for 0.5–1 min to ensure even distribution of the instillation within the lungs.

### In vivo experimental protocol

Mice (n=75) were randomly divided into five groups (n=15/group) as follows: Control, NS, NS plus air, LPS and LPS plus air. Mice in the LPS and NS groups were instilled intratracheally with LPS or NS, respectively, as described in the previous section. In the LPS plus air and NS plus air groups, LPS or NS, respectively, was instilled intratracheally with a 1-ml syringe prefilled with 0.1 ml air. Mice in the control group did not undergo any treatment. All mice were sacrificed 24 h subsequent to intratracheal instillation of LPS or NS.

### Bronchoalveolar lavage (BAL)

Mice (n=5/group) were exsanguinated and sacrificed by removal of the eyeballs following anesthesia with an intraperitoneal injection of 0.1 ml pentobarbital sodium (50 mg/kg). Following surgical isolation of the trachea, mice were intubated with a 24-gauge cannula. The lungs were flushed with 0.9% NS in 0.2-ml increments. Recovery of BAL was identical for all experimental groups and the recovery rate was 87±2%. The BAL fluid was centrifuged for 5 min at 300 × g, and the supernatant was analyzed to obtain the biochemical index. Levels of lactate dehydrogenase (LDH), alkaline phosphatase (ALP) and total protein were assessed with commercial reagent kits (Nanjing Jiancheng Bioengineering Institute, Nanjing, China) and interleukin-8 (IL-8) levels were determined with a commercial, specific ELISA kit (R&D Systems, Minneapolis, MN, USA), used in accordance with the manufacturer’s instructions. The pellet was resuspended in 1 ml phosphate-buffered saline with 1% bovine serum albumin and 0.1% sodium azide, and a 10-μl aliquot was used to count cells. Cell viability was assessed by trypan blue staining (Sigma). In addition, cytospun cells were prepared for Wright’s staining and differential cell counting using a cytocentrifuge (Academy of Military Medical Sciences, Beijing, China).

### Lung wet/dry weight ratio

Mice (n=5/group) were exsanguinated and sacrificed by removal of the eyeballs. A median sternotomy was performed, and the lungs of each mouse were excised. The wet weight of the exsanguinated whole lungs was measured with an electronic balance, and the lungs were then oven-dried at 60°C for 72 h prior to the dry weight being recorded. The wet-to-dry weight ratio was then calculated.

### Lung histology

Mice (n=5/group) were exsanguinated and sacrificed by removal of the eyeballs. The tracheas of the mice were exposed and cannulated with PE-90 tubing, the chests were opened and the lungs were removed and filled with 10% buffered formalin at an airway pressure of 20 cm H_2_O for 30 min. Paraffin embedding was performed with the lungs oriented in a prone position, and 5-μm sections were cut for hematoxylin and eosin staining. A lung injury scoring method was utilized to quantify changes in lung architecture, as evidenced by light microscopy. The following variables were used to assess the degree of microscopic injury: alveolar and interstitial edema, neutrophil infiltration and hemorrhage. Each variable was graded according to the severity of injury: no injury, 0; injury to 25% of the field, 1; injury to 50% of the field, 2; injury to 75% of the field, 3; and diffuse injury, 4 (16). The samples were analyzed based on a scaled grading system by a pathologist who was blinded to the experimental protocol and the sampling region. Three slides from each lung sample were randomly screened and the mean was taken as the representative value of the sample.

### Statistical analysis

Statistical analysis was conducted using the SPSS predictive analytics software 18.0 package (SPSS, Inc., Chicago, IL, USA), and all data are expressed as the mean ± standard error of the mean. One-way analysis of variance followed by Bonferroni (equal variances) or Dunnett’s T3 (heteroscedasticity) post hoc test were performed to determine the statistical significance between indicated groups. P<0.05 was considered to indicate a statistically significant difference.

## Results

### BAL fluid biochemical analysis

**Figure 1:**
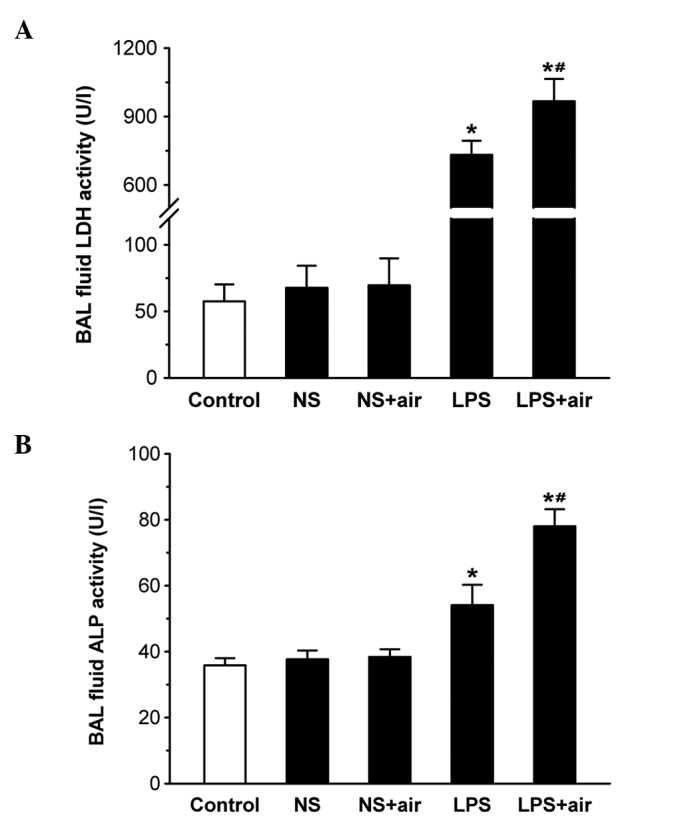
Biochemical evaluation of BAL fluid. (A) LDH and (B) ALP activities. ^*^P<0.05 compared with the control group; ^#^P<0.05 compared with the LPS group. Data are expressed as the mean ± standard error of the mean (n=5). BAL, bronchoalveolar lavage; LDH, lactate dehydrogenase; ALP, alkaline phosphatase; NS, normal saline; LPS, lipopolysaccharide.

**Figure 2:**
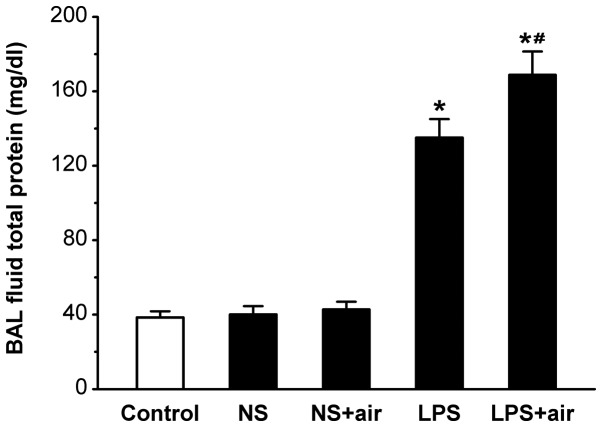
BAL fluid total protein concentrations. ^*^P<0.05 compared with the control group; ^#^P<0.05 compared with the LPS group. Data are expressed as the mean ± standard error of the mean (n=5). BAL, bronchoalveolar lavage; NS, normal saline; LPS, lipopolysaccharide.

LDH and ALP activities and total protein concentration in the BAL fluid were used as the indicators of cell injury (Fig. 1 and 2). BAL fluid LDH and ALP activities were generally consistent with the degree of cell injury: Cell injury enhances the permeability of the alveolar-capillary barrier, leading to an increased protein concentration in BAL fluid. No significant differences were observed in BAL fluid LDH and ALP activities and total protein concentrations between the sham-treated (NS plus air and NS) and control groups. BAL fluid LDH and ALP activities and total protein concentrations were significantly increased in the LPS plus air and LPS groups compared with the control group (all P<0.05). These three indices were significantly increased in the LPS plus air group compared with the LPS group (all P<0.05). The results indicated that the instilled air aggravated LPS-induced cell injury.

### BAL fluid differential cell counting

**Figure 3:**
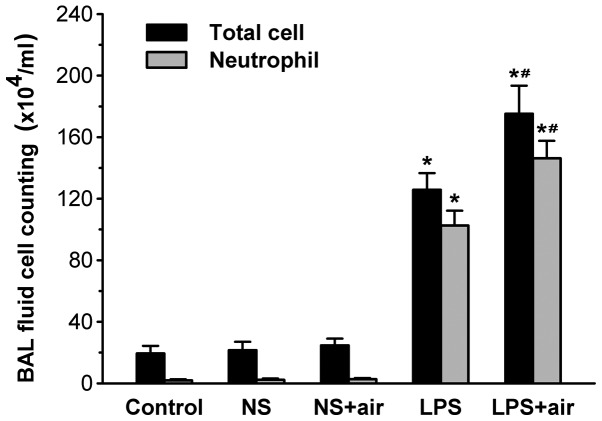
BAL fluid differential cell counting. ^*^P<0.05 compared with the control group; ^#^P<0.05 compared with the LPS group. Data are expressed as the mean ± standard error of the mean (n=5). BAL, bronchoalveolar lavage; NS, normal saline; LPS, lipopolysaccharide.

BAL fluid differential cell counting was used to evaluate the number and types of migrated cells, and to further indicate the category and extent of pulmonary inflammation (Fig. 3). No significant differences were observed in the total cell and neutrophil numbers between the sham-treated (NS plus air and NS) and control groups. The total cell and neutrophil numbers were significantly increased in the LPS plus air and LPS groups compared with the control group (all P<0.05). Furthermore, the total cell and neutrophil numbers were significantly increased in the LPS plus air group compared with the LPS group (all P<0.05). These results indicated that the instilled air promoted LPS-induced neutrophil infiltration into the lungs.

### Lung wet/dry weight ratio

**Figure 4:**
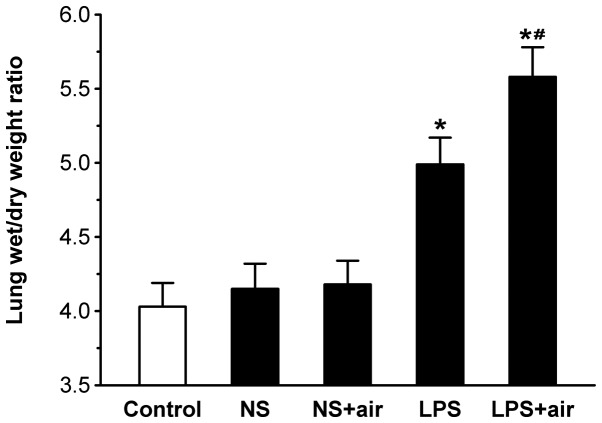
Lung wet/dry weight ratio. ^*^P<0.05 compared with the control group; ^#^P<0.05 compared with the LPS group. Data are expressed as the mean ± standard error of the mean (n=5). LPS, lipopolysaccharide; NS, normal saline.

Lung wet/dry weight ratio is used as an indicator of pulmonary edema (Fig. 4). No significant differences were observed in the lung wet/dry weight ratios between the sham-treated (NS plus air and NS) and control groups. The lung wet/dry weight ratios were significantly increased in the LPS plus air and LPS groups compared with the control group (all P<0.05). The lung wet/dry weight ratio was significantly increased in the LPS plus air group compared with the LPS group (P<0.05). These results indicated that the instilled air exacerbated LPS-induced pulmonary edema.

### Lung histology

**Figure 5:**
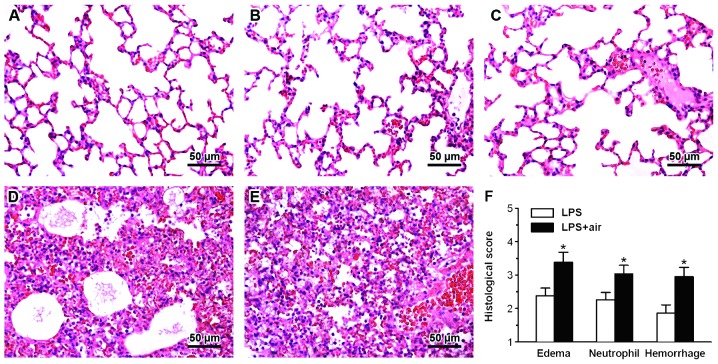
Histological changes in LPS-induced acute lung injury 24 h subsequent to stimulation (hematoxylin and eosin staining; magnification, ×400; scale bar, 50 μm). (A) Control group; (B) NS group; (C) NS plus air group; (D) LPS group; (E) LPS plus air group; (F) histological score. ^*^P<0.05 compared with the LPS group. Data are expressed as the mean ± standard error of the mean. LPS, lipopolysaccharide; NS, normal saline.

No marked differences were observed in the lung histology between the sham-treated (NS plus air and NS) and control groups, indicating that intratracheal instillation of NS with or without air did not result in acute lung inflammation (Fig. 5A–C). There were different degrees of fluid accumulation, neutrophil infiltration, congestion and hemorrhage in the LPS plus air and LPS groups compared with the control group. There was more protein-rich fluid, and a greater number of neutrophils and erythrocytes in the alveoli of mice in the LPS plus air group compared with the LPS group (Fig. 5D and E). Edema, neutrophil infiltration and hemorrhage scores were significantly increased in the LPS plus air group compared with the LPS group (all P<0.05; Fig. 5F). These results indicated that the instilled air aggravated LPS-induced pathological changes.

### BAL fluid IL-8 concentration

**Figure 6:**
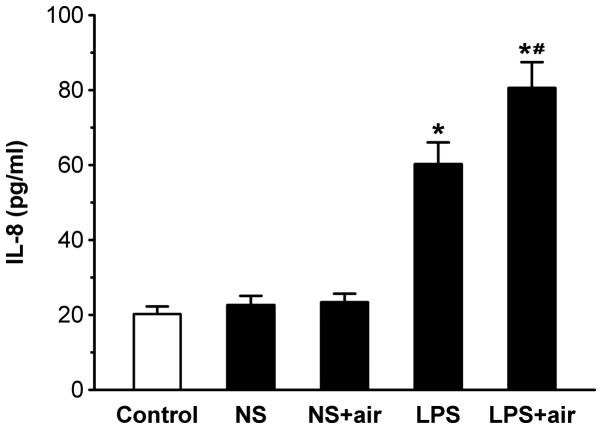
IL-8 release in BAL fluid. ^*^P<0.05 compared with the control group; ^#^P<0.05 compared with the LPS group. Data are expressed as the mean ± standard error of the mean (n=5). IL, interleukin; BAL, bronchoalveolar lavage; LPS, lipopolysaccharide; NS, normal saline.

BAL fluid IL-8 concentration was assessed due to its important role in the pathogenesis of ALI (Fig. 6). No significant differences were observed in the IL-8 levels between the sham-treated (NS plus air and NS) and control groups. Levels of IL-8 were significantly increased in the LPS plus air and LPS groups compared with the control group (all P<0.05). IL-8 levels were significantly increased in the LPS plus air group compared with the LPS group (P<0.05). These results indicated that instilled air increased LPS-induced IL-8 release.

## Discussion

The improvement of intratracheal instillation procedures may facilitate the establishment of an optimal animal model of ALI in order to further reveal the cellular and molecular pathogenesis of ALI. In the present study, the effect of instilling air by trans-tracheal intratracheal instillation on an LPS-induced murine model of ALI was investigated. The results demonstrated that trans-tracheal intratracheal air instillation promoted LPS-induced ALI, as shown by the more severe acute pulmonary inflammation and increased IL-8 release.

Alveolar epithelial and vascular endothelial injury, neutrophil infiltration and increased membrane permeability, leading to pulmonary edema, are involved in the pathogenesis of ALI/ARDS (1). In ALI, binding of LPS to Toll-like receptors on lung cells initiates acute lung inflammation (17). Chemokines are secreted from the stimulated pulmonary epithelium and alveolar macrophages recruit neutrophils into airspaces via the alveolar-capillary barrier (18–20). Activated neutrophils release a variety of mediators, including proteases, reactive oxygen species, histones and peptides, which cause vascular endothelial and alveolar epithelial injuries (21). The subsequent increase in the permeability of the alveolar-capillary barrier leads to the extravascular accumulation of protein-rich edema fluid (22). Disruption of the capacity for fluid clearance and surfactant production due to pulmonary epithelial injury also aggravates pulmonary edema (23). In the present study, instilled air promoted LPS-induced ALI, as evidenced by the more severe acute pulmonary inflammation, including cell injury, neutrophil infiltration and permeability pulmonary edema.

Neutrophils have been indicated to be involved in the pathogenesis of ALI/ARDS, and IL-8 has been identified as the main chemotactic factor for neutrophils in the lung fluid of patients with ALI/ARDS (24–26). IL-8 may enhance the migratory activity of neutrophils and induce migration through the alveolar-capillary barrier, resulting in an accumulation of neutrophils in the alveolar spaces. Therefore, IL-8 levels reflect the severity of ALI in animals and humans. In this study, BAL fluid IL-8 levels were used to further evaluate the influence of instilled air on LPS-induced ALI. Instilled air increased LPS-induced IL-8 release, indicating that instilled air promotes LPS-induced ALI by increasing IL-8 levels. It has been demonstrated that alveolar epithelia are capable of producing a higher level of IL-8 than bronchial epithelia under the stimulation of LPS (13). However, it is possible that the instilled air resulted in delivery of LPS to the alveolar and bronchial epithelial cells in different proportions. Instilled air may drive the discharge of LPS from the syringe, delivering an enhanced level of LPS into the alveolar spaces and resulting in greater LPS exposure in the alveolar epithelia, increased IL-8 release and more severe acute pulmonary inflammation.

This study demonstrates that instilled air promotes LPS-induced ALI. To the best of our knowledge, this is the first study to reveal the role of intratracheal air instillation in an LPS-induced experimental animal model of ALI. Instilled air may be used to improve the method of intratracheal instillation and establish a more reliable experimental animal model of ALI. This may enable the further elucidation of the molecular pathogenesis of ALI/ARDS. The instilled air may deliver more LPS into the alveolar spaces, leading to more severe acute pulmonary inflammation. The results of this study may provide guidance for the establishment of other animal models and for approaches to improve drug delivery.
